# A comparison of the clinical effect of two fixation methods on Hoffa fractures

**DOI:** 10.1186/s40064-016-2861-6

**Published:** 2016-07-25

**Authors:** Yi Xu, Heng Li, Hong-hang Yang, Zhi-jun Pan

**Affiliations:** 1 Department of Orthopaedics, The First People’s Hospital of Huzhou (The First Affiliated Hospital of Huzhou Teachers College), Zhejiang University, Huzhou, 313000 Zhejiang China; 2 Department of Orthopaedic Surgery, The Second Affiliated Hospital, School of Medicine, Zhejiang University, Hangzhou, 310009 Zhejiang China

**Keywords:** Hoffa fracture, Femoral intercondylar notch, Open reduction and internal fixation

## Abstract

**Introduction:**

Hoffa fractures are rare and difficult to treat for orthopaedic surgeons. The mechanism of injury of Hoffa fracture is still unknown and the operation approch and fixation method are still controversial. The aim of this study is to compare the clinical effect between two fixation methods on Hoffa fractures.

**Case description:**

From April 2004 to July 2013, we treated eleven patients (new method group) with Hoffa fracture using the new fixation method (fixed with intercondylar screw and crossed screws) and sixteen patients (traditional method group) using the traditional fixation method (fixed with anteroposteriorly placed screws). All documents from their admission until the last followup in December 2015 were reviewed, data regarding complications collected and results were evaluated using the Knee Society Score.

**Discussion and Evaluation:**

After an average follow-up period of 27.1 months (range 24–32 months), all fractures had healed. The average healing time of the new method group was 11.36 weeks (range 9–14 weeks) and the average healing time of the traditional method group was 11.88 weeks (range 9–14 weeks). According to the Knee Society Score, the average score of the new method group was 176.36 points (range 125–199 points), and the average score of the traditional method group was 171.19 points (range 148–197 points). Statistical analysis (*t* test, *t* = 0.76, *P* > 0.05) showed that the difference of both the healing time (*t* test, *t* = 0.94, *P* > 0.05) and the score between these two groups was not significant.

**Conclusions:**

These results indicate that the new fixation method for Hoffa fracture is as effective as the traditional method and may provide a new way to treat Hoffa fractures.

## Background

Hoffa fractures are rare injuries that are first described by Hoffa (1904). Nonoperative treatment of Hoffa fractures usually results in poor outcomes (Ostermann et al. 1994; Lewis et al. 1989; Borse et al. 2010; Jarit et al. 2006). So anatomic reduction and rigid internal fixation of Hoffa fractures are very important to allow early knee motion and help knee functional recovery. Since April 2004, we treated Hoffa fractures using a new fixation method (fixed with intercondylar screw and crossed screws) and achieved good results (Xu et al. 2013). We report our results of treating Hoffa fractures by two fixation methods and review the literatures concerning the treatment of Hoffa fractures.

## Patients and methods

**Fig. 1:**
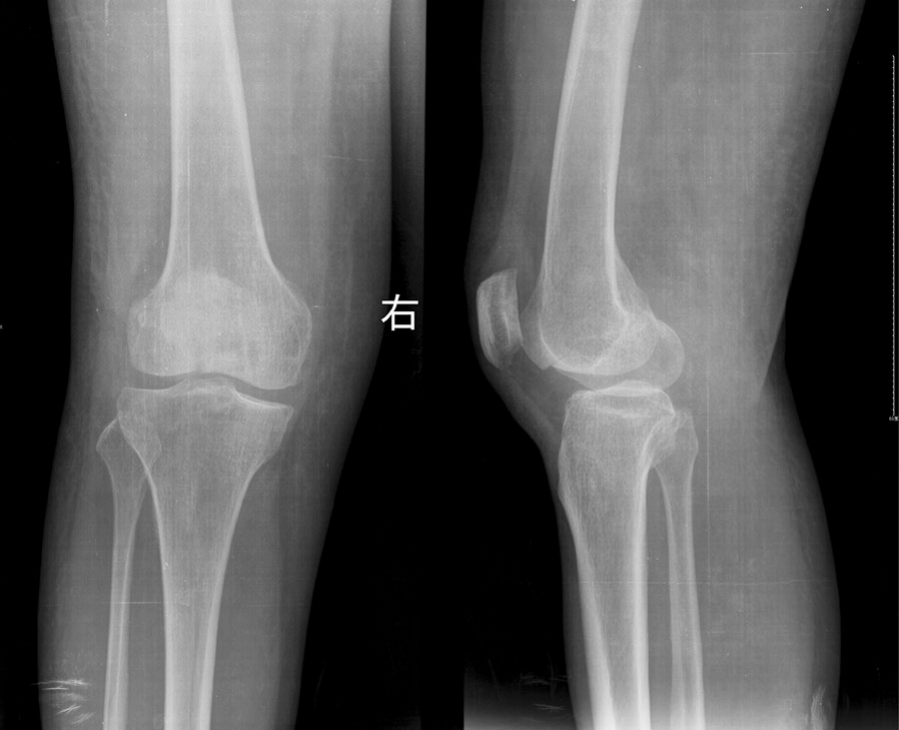
Anteroposterior and lateral radiographs show Hoffa fracture (type III) of the lateral femoral condyle

**Fig. 2:**
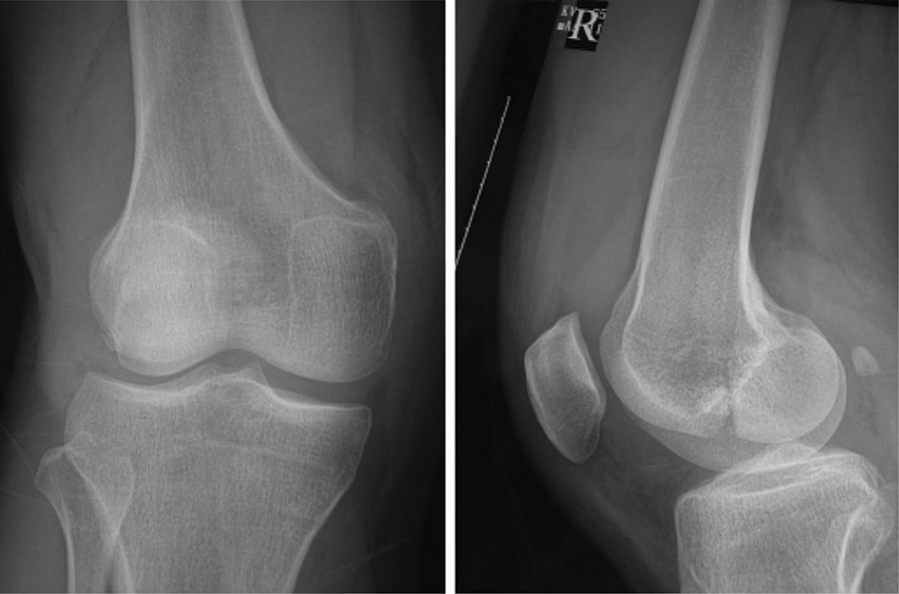
Anteroposterior and lateral radiographs show Hoffa fracture (type I) of the lateral femoral condyle

In a retrospective study, twenty-seven patients who underwent open reduction and internal fixation for unicondylar Hoffa fractures between April 2004 to July 2013 were enrolled. Eleven patients (new method group) were treated by the new fixation method (fixed by intercondylar screw and crossed screws). There were eight males and three females, aged from 23 to 48 years (average 38.45 years). Eight fractures were lateral, and three were medial. The mechanism of injury was a motor vehicle accident in all patients. According to Letenneur classification (Letenneur et al. 1978), seven fractures were type I and four were type III. Preoperative X-rays of one of ten cases were shown in Fig. 1. Sixteen patients (traditional method group) were treated by the traditional method (fixed by anteroposteriorly placed screws). There were eleven males and five females, aged from 20 to 51 years (average 38.19 years). Twelve fractures were lateral, and four were medial. The mechanism of injury was a motor vehicle accident in fourteen patients and a fall in two patients. According to Letenneur classification, ten fractures were type I and six were type III. Preoperative X-rays of one of sixteen cases were shown in Fig. 2. According to *F* test, the difference of both age (*F* = 1.21, *P* > 0.05) and type (*F* = 1.19, *P* > 0.05) between these two groups was not significant.

### Operative procedure

**Fig. 3:**
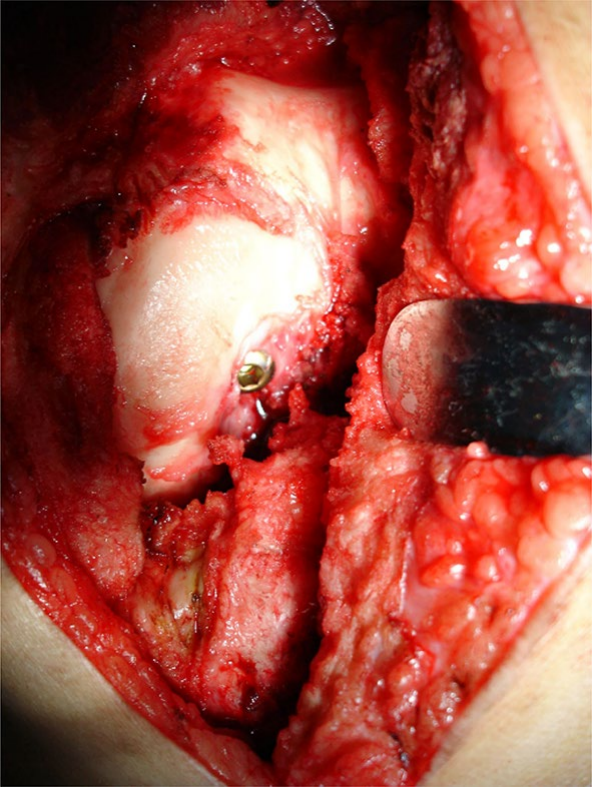
The screw is inserted from the femoral intercondylar notch

**Fig. 4:**
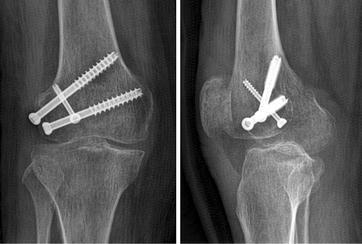
Anteroposterior and lateral radiograph shows the fracture is fixed with three screws, one screw is inserted from the femoral intercondylar notch

Under general anaesthesia, an anterolateral (or anteromedial) incision is made, with lateral (or medial) parapatellar release and medial (or lateral) dislocation of the patella, the fractured condyle is exposed. With full flexion of the knee, the fracture can be exposed clearly. Using a bone repositioning clamp to hold the fracture fragment, the fracture can be reduced by traction of the calf and slow stretching of the knee. Then the knee is flexed again and the fracture is preliminarily fixed with two Kirschner wires. The fracture is fixed by three cancellous screws. One screw (3.5- or 4.5-mm) is inserted from the femoral intercondylar notch (Fig. 3) and is directed anterolaterally (or anteromedially) to thread through the fractured condylar fragment. The other two screws (6.5-mm) are inserted from the non-articular lateral (or medial) surface of the fractured condylar fragment, and are directed medially (or laterally) to thread through the fractured condylar fragment (Fig. 4). One of these two screws is placed in a certain angle to reach the opposite femoral shaft. Two sides of screws are crossed.

**Fig. 5:**
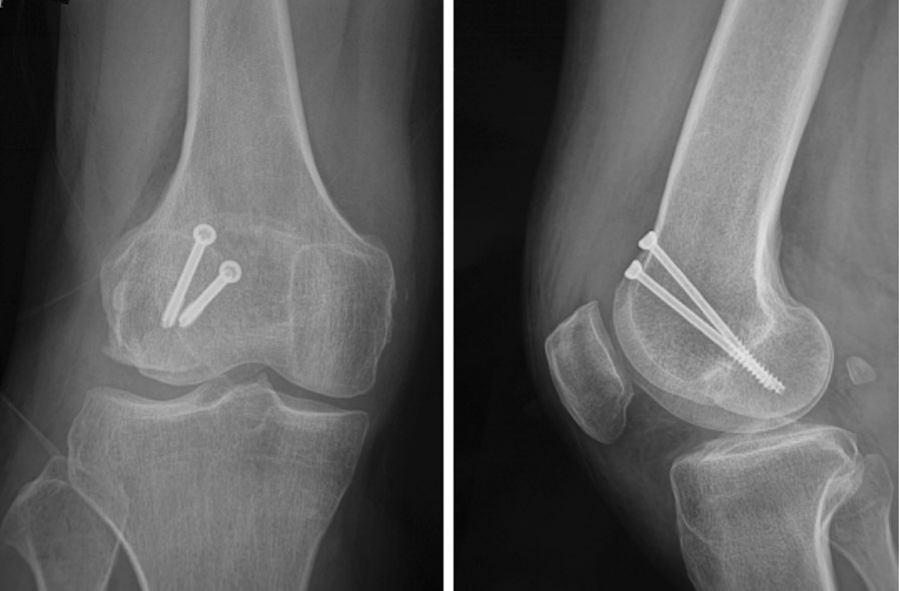
Anteroposterior and lateral radiograph shows the fracture is fixed with two anteroposterior parallel screws

The traditional method use the same approach, but the fracture is fixed with two parallel screws. The screws are inserted from the non-articular area just proximal to the patellafemoral joint with an anteroposterior direction to engage the fractured condylar fragment (Fig. 5).

Postoperatively, all patients began unrestricted range of motion. Initial weight-bearing status was limited, but all patients were allowed full weight bearing within 3 months.

### Statistical analysis

Statistical analyses were performed using SPSS version 13.0 (SPSS Inc, Chicago, IL) for Windows. Data are shown as mean ± standard error of the mean, except where indicated otherwise. The Student’s *t* test was used to compare continuous variables. The Chi square test was used to evaluate the differences in clinical outcomes between potential associated factors. *P* values below 0.05 were accepted for statistical significance.

## Results

After an average follow-up period of 27.07 months (range 24–32 months), all of the fractures healed both clinically and radiologically, without deep infection, nonunion, malunion, osteonecrosis and hardware removals. The healing time of the new method group was 11.36 ± 1.36 weeks (range 9–14 weeks) and the average healing time of the traditional method group was 11.88 ± 1.41 weeks (range 9–14 weeks). According to the Knee Society Score, the score of the new method group was 176.36 ± 20.65 points (Table 1), and the score of the traditional method group was 171.19 ± 15.04 points (Table 2). Statistical analysis showed that the difference of both the healing time (*t* test, *t* = 0.94, *P* > 0.05) and the score (*t* test, *t* = 0.76, *P* > 0.05) between these two groups was not significant.

**Table 1:** Information and results of the patients in the new method group

Case	Gender	Age (years)	Lateral or medial	Type	Results			
					Time of fractures healing (weeks)	Objective score (points)	Functional score (points)	Total score (points)
1	F	36	Lateral	I	9	76	80	156
2	M	23	Lateral	I	12	94	100	194
3	M	45	Lateral	I	11	89	80	169
4	M	48	Medial	I	11	88	100	188
5	M	32	Medial	III	10	88	90	178
6	F	44	Medial	I	13	89	97	186
7	M	46	Lateral	III	12	65	60	125
8	M	40	Lateral	III	11	90	90	180
9	F	42	Lateral	I	14	95	90	185
10	M	27	Lateral	I	11	99	100	199
11	M	40	Lateral	III	11	90	90	180

**Table 2:** Information and results of the patients in the traditional method group

Case	Gender	Age (years)	Lateral or medial	Type	Results			
					Time of fractures healing (weeks)	Objective score (points)	Functional score (points)	Total score (points)
1	F	36	Lateral	I	9	83	95	178
2	M	49	Medial	I	11	80	75	155
3	M	26	Lateral	I	11	80	82	162
4	M	42	Lateral	I	14	95	99	194
5	M	32	Medial	III	10	91	94	185
6	M	20	Lateral	I	12	75	87	162
7	M	46	Lateral	III	11	71	82	153
8	F	44	Medial	III	14	78	80	158
9	M	30	Lateral	I	13	85	95	180
10	F	33	Lateral	I	12	78	70	148
11	M	51	Lateral	III	13	85	84	169
12	M	46	Medial	I	11	98	99	197
13	F	39	Lateral	I	13	79	96	175
14	M	46	Lateral	III	13	85	91	176
15	F	35	Lateral	III	12	90	97	187
16	M	36	Lateral	I	11	80	80	160

## Discussion

Hoffa fractures are unstable intraarticular fractures, and lateral Hoffa fractures are more common than medial ones (Ostermann et al. 1994; Xu et al. 2013; Letenneur et al. 1978; Nork et al. 2005; Dhillon et al. 2012; Biau and Schranz 2005). Bicondylar Hoffa fractures are rare (Papadopoulos et al. 2004; Calmet et al. 2004). In our study, there are twenty lateral Hoffa fractures and seven medial Hoffa fractures, which is similar to the literatures. The mechanism of injury of Hoffa fracture is still unknown. Some authors consider direct impact with the knee in a flexed position as the mechanism of injury, while others think that the fracture is caused by simultaneous vertical shear and twisting forces (Lewis et al. 1989; Papadopoulos et al. 2004). In initial anteroposterior and lateral radiographs, Hoffa fractures, especially when nondisplaced, are sometimes difficult to be observed. So a CT scan or MRI scan is necessary to define the fractures (Nork et al. 2005).

Hoffa fracture is classified as a type 33-B3 fracture by the Orthopaedic Trauma Association. But this classification provides little information of prognosis and treatment. Letenneur et al. (1978) reported that they divided Hoffa fractures into three types based on the distance of the fracture line from the posterior cortex of the femoral shaft. In a cadaveric study, Lewis et al. (1989) finded that in Type I and Type III Hoffa fractures there are some soft tissue elements attached to the fractured condylar fragment to provide blood supply to this fragment. But in Type II Hoffa fractures there was no soft tissue elements attached to the fractured condylar fragment.

Hoffa fractures are intraarticular fractures. Most of them need surgical open reduction and internal fixation to achieve good outcome (Lewis et al. 1989; Letenneur et al. 1978; McDonough and Bernstein 2000; Manfredini et al. 2001). But the operation approach and fixation method are still been improving. It is generally accepted that screw fixation is a good fixation method for treating Hoffa fractures. In Type I and Type III Hoffa fractures, an anterolateral (or anteromedial) incision is usually used, and two anteroposteriorly placed lag screws are inserted from the non-articular area just proximal to the patella-femoral joint to engage the fractured condylar fragment. One screw is inserted into the femoral shaft to provide rotational stability (Ostermann et al. 1994). In Type II Hoffa fractures, because the fracture line is near the articular cartilage of the posterior condyle, a posterior approach and two posteroanteriorly placed lag screws may be a good choice (Medvecky and Noyes 2005; Tan et al. 2013). However, the screws are inserted through the articular surface, so the screw heads should be countersunk.

In our study, the new fixation method use only three screws. One screw is inserted into the femoral intercondylar notch to avoid affecting the knee function by the articular surface defect and decreases the risk of osteoarthritis. The other two screws are inserted from the non-articular lateral (or medial) surface of the fractured condylar fragment, two sets of screws are crossed, so both sides of the fracture lines are completely compressed. Three screw heads are shaped into a triangle, which can protect against shear and twisting forces and obtain better stabilization. We also find that this new method is more suitable for type III Hoffa fractures. Because in type III Hoffa fractures, the fracture line is oblique and the fracture fragment in the intercondylar notch is big enough to insert a screw. But in type I Hoffa fractures, the fracture line is vertical and the screw entry point must be closed to the weight-bearing area of the articular cartilage.

Jarit et al. (2006) reported that Lag screws placed in a posteroanterior direction were more stable than anteroposteriorly placed lag screws. In our new method, the screws are not placed in a posteroanterior direction, which is an imperfection. But the crossed screws are more rigid than the parallel screws, especially in resisting torsional stresses (Friedman et al. 1994).

Becker et al. (2000) had a cadaveric study to compare the stiffness and load to failure among 3.5-mm cortical lag screws, 4.5-mm cortical lag screws, and 6.5-mm cancellous screws to fix experimentally created Hoffa fractures. There was no difference in stiffness between these three groups, but compared with 3.5-mm screws, the load to failure was significantly higher for 6.5-mm screws. We inserted a 3.5-mm or 4.5-mm screw from the femoral intercondylar notch to decrease articular cartilage defects. But the other two screws were 6.5-mm screws to bear more loads.

However, in our study there is no direct evidence shows that the stability of the new method is better than the traditional method, which needs a further mechanical study. And the difference of the risk of osteoarthritis between these two methods needs a long-term follow-up.

In a summary, the results of our study show that the new fixation method for Hoffa fracture is as effective as the traditional method and this new fixation method may be more suitable for type III Hoffa fractures.
